# Advances in engineering and applications of synthetic phase-separated membraneless organelles in biotechnology

**DOI:** 10.1016/j.synbio.2026.01.007

**Published:** 2026-01-22

**Authors:** Manman Sun, Alex Xiong Gao, Bin Ye, Yimeng Zhao, Rodrigo Ledesma-Amaro, Jin Gao, Peng Wang

**Affiliations:** a Key laboratory of high magnetic field and Ion beam physical biology, Hefei Institutes of Physical Science, Chinese Academy of Sciences, Hefei, 230031, China; b Department of Bioengineering and Imperial College Centre for Synthetic Biology, Imperial College London, London, SW7 2AZ, UK; c Institute of Hefei Artificial Intelligence Breeding Accelerator, Hefei, 230000, China; d Division of Life Science, The Hong Kong University of Science and Technology, 999077, Hong Kong, China; e Department of Neurobiology and Cellular Biology, Xuzhou Medical University, Xuzhou, 221004, Jiangsu, China

**Keywords:** Synthetic membraneless organelles, Phase-separated scaffolds, Metabolic engineering, Protein engineering, Functional biomaterials

## Abstract

Membraneless organelles (MLOs) formed through liquid-liquid phase separation (LLPS) constitute crucial dynamic microenvironments within cells, capable of selectively concentrating specific molecules and regulating biochemical reactions. Based on the working mechanisms of natural MLOs, researchers have designed and constructed various synthetic MLOs. These MLOs have been applied in regulating enzyme activity, optimizing metabolic pathways, regulating gene expression, producing recombinant proteins, and developing functional biomaterials. Here, we systematically summarized the design strategies, characterization techniques, and client protein recruitment methods for synthetic MLOs, and categorically reviewed their application progress in the biotechnology field. We also discussed current challenges faced in the practical applications of synthetic MLOs and future research directions. This review aims to provide theoretical guidance and practical reference for the design and application of LLPS-driven synthetic MLOs, thereby promoting their innovative development in synthetic biology and biotechnology.

## Highlights

• Reviews major scaffold types for constructing synthetic membraneless organelles via LLPS.
• Details strategies for client protein recruitment and dynamic control within synthetic organelles.
• Summarizes characterization methods revealing phase behavior and material properties of MLOs.
• Categorizes diverse applications of synthetic MLOs in biotechnology.
• Discusses challenges and future directions for the construction and application of MLOs.

## Graphical abstract

**Figure:**
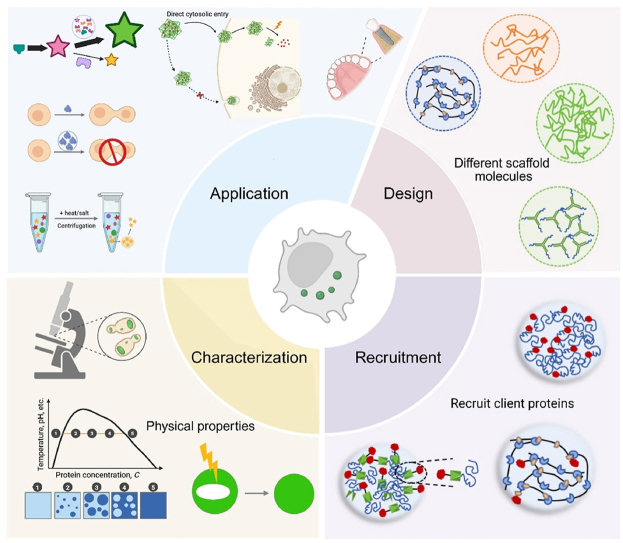

## 1 Introduction

Membraneless organelles (MLOs), also known as biomolecular condensates, are dynamic, membraneless cellular compartments that facilitate the spatial and temporal organization of biomolecules within cells [1]. Unlike traditional membrane-bound organelles, MLOs are primarily formed through liquid-liquid phase separation (LLPS), a process driven by weak multivalent interactions, including electrostatic forces, π-π stacking, and hydrophobic interactions [2]. These interactions enable MLOs to selectively recruit specific proteins, nucleic acids, and other biomolecules, creating specialized microenvironments that modulate biochemical reactions by concentrating reactants and tuning molecular interactions. The dynamic and reversible assembly of MLOs further enables them to disassemble and reassemble in response to cellular conditions, which is essential for regulating processes like gene expression, signal transduction, and stress responses [3].

Inspired by the functionality of natural MLOs, engineered or synthetic MLOs have been developed as programmable microreactors. The construction of these artificial compartments typically leverages molecules capable of undergoing phase separation, such as intrinsically disordered proteins (IDPs), specifically folded proteins, and nucleic acids [1,4]. To further enhance controllability, stimuli-responsive modules (e.g., light-, pH-, or small-molecule-sensitive switches) have been integrated into these systems, enabling spatiotemporal regulation of condensate formation and dissolution under defined environmental conditions [[5], [6], [7], [8]]. To date, synthetic MLOs have been successfully constructed in mammalian cells and a variety of microbial systems, including *Saccharomyces cerevisiae*, *Escherichia coli*, *Bacillus subtili*s, and *Corynebacterium glutamicum* [[8], [9], [10], [11], [12]]. They have demonstrated great potential in regulating metabolic flux, enhancing product synthesis, modulating cellular behaviors, and even mitigating the cytotoxic effects of toxic protein expression [1,8,10,13].

Here, we provide a comprehensive overview of recent advances in the design and application of synthetic phase-separated MLOs. We summarize the construction strategies utilized for constructing these synthetic compartments and the analytical methods used to characterize their properties. Furthermore, we review their current applications in biotechnology. The challenges faced and potential future research directions are also specifically discussed.

## 2 The design and construction of synthetic membraneless compartments

The construction of synthetic MLOs typically involves two fundamental components: scaffold molecules and client molecules [4,14]. Scaffold molecules drive phase separation through multivalent and often weak, reversible interactions, which determine key material properties of MLOs, such as fluidity, viscoelasticity, stability, saturation concentration (*C*_sat_), and selective permeability [15]. Client molecules are selectively recruited into the MLOs via specific interactions with the scaffold, imparting functionality to the synthetic compartments [8,16]. The modular and orthogonal nature of scaffold–client systems allows for flexible and precise engineering, enabling synthetic MLOs to be customized for diverse applications [1,4]. In the following sections, we summarize representative scaffold classes and their engineering considerations (Table 1).

**Table 1:** Comparison of different scaffold molecules.

	Intrinsically disordered proteins	Multivalent modular domains	Nucleic acids
Key features	Low-complexity, disordered sequences	Programmable, orthogonal binding pairs	Sequence-programmable multivalency via repeats/aptamer arrays or sticky ends/branching.
Driving forces	Multivalent weak interactions: electrostatics, π–π/cation–π, hydrophobic, hydrogen bonding	Specific multivalent interactions between modular pairs	Base pairing/stacking, multivalent protein–RNA binding, programmable DNA interactions.
Key points^a^	Optimize sequence features, increase valency, use programmable artificial IDPs, integrate responsive modules	Increase valency of both partners, tune stoichiometry and affinity, design orthogonal pairs	Increase repeat units, embed multiple aptamer arrays
Advantages	Strong LLPS capacity, performs well across diverse systems	More stable interactions, smaller tags minimize client perturbation, highly tunable	Highly programmable and orthogonal, small recruitment tags on clients minimize perturbation
Limitations	Large molecular weight, potential functional interference to client proteins, disease associations in some cases	High affinity leads to gelation, sensitive to stoichiometry and competition	RNA stability, potential crosstalk with host RNAs, stoichiometry-sensitive
Representative scaffolds	FUS, RGG, Ddx4	SH3–PRM, SUMO–SIM	RNA TEARS, Y-shaped DNA nanostars

a Key points to enhance phase separation capacity.

### 2.1 IDPs as scaffold molecules

**Fig. 1:**
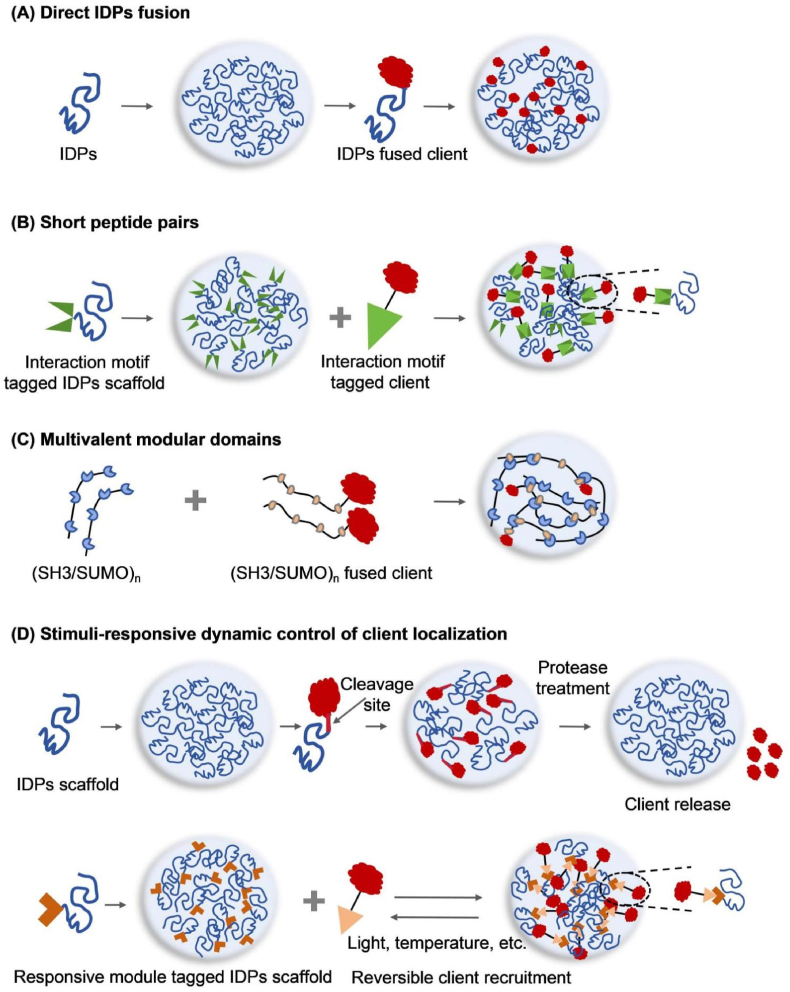
**Strategies for MLOs construction and client molecule recruitment.** (A) Direct fusion of IDPs (e.g., RGG domain, FUS family proteins) to client proteins; (B) Client recruitment via compact, short interacting peptide pairs (C) Use of multivalent modular domains (e.g., SUMO/SIM, SH3/PRM); to recruit clients; (D) Dynamic control of client localization and release.

Intrinsically disordered proteins (IDPs) are widely used scaffolds for constructing synthetic MLOs. Lacking stable tertiary structure and enriched in low-complexity sequences (LCSs), IDPs undergo LLPS via multivalent, weak interactions, including electrostatics, π–π and cation–π contacts, hydrogen bonding, and hydrophobic association, yielding liquid-like condensates with rapid molecular exchange [1,17,18] (Fig. 1). Natural IDPs have been extensively utilized in synthetic MLOs construction. For example, the prion-like low complexity domains (PLCDs) of FUS family proteins exhibit robust phase separation, forming liquid-like condensates in vitro and in vivo that can be spatiotemporally controlled [8,19]. The RGG domain of LAF-1 also demonstrates robust phase separation properties across different systems [10,12,20]. Rational modulation of these natural IDPs, such as tuning the ratio and patterning of aromatic-to-charged residues in sequences like Ddx4 or inserting orthogonal functional modules, enables precise control of droplet permeability, stability, and enzymatic sequestration capability [19,21]. However, these natural IDPs scaffolds often exhibit intrinsic complexity and heterogeneity that complicate their predictability and tunability in engineered contexts, due to environmental sensitivity and potential aggregation tendencies.

To overcome these limitations, artificial IDPs (A-IDPs) and de novo designed polypeptides have been engineered to enhance programmability, stability, and modularity. These designs often employ repeat-based “sticker–spacer” architectures, allowing rational tuning of phase separation parameters such as saturation concentration, droplet viscoelasticity, and client recruitment specificity. For instance, stacking short peptide motifs (e.g., FW1, RGYGSPDG) yields programmable condensates that boost 2′-fucosyllactose (2′-FL) biosynthesis and improve ncAA translation selectivity across hosts [11]. GRGDSPYS-based A-IDPs exhibit well-predicted LLPS both in vitro and in living cells, demonstrating tunable sequestration of enzymes and modulation of catalytic efficiency with precise control over chain length and aromatic content [22]. Additionally, tools like IUPred2A and CIDER are widely used to predict disordered regions and analyze sequence properties, providing valuable guidelines for the rational design and optimization of artificial IDPs [10,12,15].

To further enhance MLOs formation, strategies to increase valency and local concentration of IDP scaffolds have been explored. Tandem repeats of IDPs, such as multiple copies of the RGG domain of LAF-1 or the LCD of FUS, have shown a strong correlation between repeat number and phase separation efficiency [13,23,24] (Table 1). Fusing IDPs with oligomerization domains, such as the pentameric NPM1 or self-assembling protein cages like human ferritin, can significantly promote phase separation by locally concentrating scaffold molecules [19,25]. Beyond scaffold design, incorporating stimulus-responsive modules, such as light-sensitive domains (Cry2, PixD/PixE) or chemically-inducible dimerization systems, enables spatiotemporal and reversible control over synthetic MLOs assembly and disassembly, greatly expanding their functional versatility [1,8]. These approaches also help fine-tune material properties to balance permeability and client recruitment, critical for applications in cellular regulation and biocatalysis.

### 2.2 Multivalent modular domains as phase-separating scaffolds

In contrast to IDPs that rely on distributed, weak interactions, multivalent modular domains drive phase separation via defined, tunable folded domain–ligand interactions that form dense, ordered networks with higher predictability and control (Fig. 1). The interaction between SRC homology 3 (SH3) domains and proline-rich motifs (PRMs) is one of the most well-characterized scaffold systems and is widely used to study phase separation [26]. Tandem repeats of SH3 or PRM modules create dense interaction networks, enhancing condensate size, reducing the critical concentration for phase separation (*C*_sat_), and decreasing droplet fluidity [27,28]. Interactions between small ubiquitin-related modifier (SUMO) domains and SUMO interaction motifs (SIMs) serve as another robust scaffold for constructing synthetic condensates. By designing fluorescently tagged mono-, di-, and tri-valent client molecules (e.g., GFP/RFP-(SUMO)_n_ and GFP/RFP-(SIM)_n_, where n = 1,2,3), researchers have demonstrated that client valency directly regulates the partitioning and composition of condensates [14] (Table 1).

The modularity and predictable binding architecture of these scaffolds enable advanced engineering of biomolecular condensates with tailored properties. For example, logic-gated systems ("OR gate" or "AND gate") have been constructed by combining modular scaffold pairs (SH3/PRM or SUMO/SIM) with specific ligands, enabling programmable co-assembly, segregation, and crosslinking of cellular components [29]. Such systems have successfully controlled localization and interactions of cell surface proteins (CSPs) like epidermal growth factor receptor (EGFR) and death receptor 5 (DR5), facilitating precise spatial and temporal regulation of cellular signaling [29]. More recently, phase separation platforms based on modular domain interactions have been harnessed to dynamically regulate RNA localization, selectively recruiting specific RNAs into synthetic condensates to spatially control RNA processing and translation [13]. Compared to IDP-based scaffolds, multivalent modular domain scaffolds present several advantages. Their structural basis is well-characterized, facilitating their rational modification and optimization. They can be easily expressed as soluble proteins in heterologous systems, whereas IDPs often tend to aggregate [30]. Furthermore, phase separation driven by multivalent modular domains is more predictable and controllable, with a reduced risk of irreversible phase transitions such as gelation or solidification under physiological conditions [31]. This improved stability minimizes pathological risks, as many IDP-driven phase separations are associated with disease processes and may trigger undesirable cellular responses [32].

### 2.3 Nucleic acids as phase-separating scaffolds

In addition to protein-driven phase separation, nucleic acids have also been demonstrated as effective scaffolds for driving the formation of MLOs [33,34]. RNA molecules can spontaneously form condensates through specific sequence motifs, recapitulating the functions of natural MLOs within cells. RNA-based synthetic condensates demonstrate selective recruitment and compartmentalization of proteins and molecules through aptamer-protein interactions or sequence recognition, offering unique programmability and addressability. A representative example is the transcriptionally engineered addressable RNA solvent droplets (TEARS) constructed by Guo et al. in *E. coli* [35]. TEARS utilizes a 47-repeat CAG trinucleotide RNA sequence (rCAG) as the phase separation domain, engineered with RNA aptamers like MS2 hairpins to recruit RNA-binding proteins fused with client enzymes. The resulting droplets exhibit multilayered liquid-like morphology with high RNA enrichment and dynamic regulation capability, enabling metabolic engineering applications such as enhanced enzymatic reaction efficiency and controlled metabolic flux via spatial organization. Increasing the length and copy number of CAG repeats substantially influences condensate number, size, density, and partition coefficient, allowing precise tuning of droplet properties [35]. Moreover, RNA scaffolds can be dynamically regulated by external stimuli such as changes in magnesium ion concentration, further expanding their utility in regulating cellular functions.

DNA has also been employed to create synthetic phase-separated condensates. DNA molecules can be designed with specific sequences and structures to promote self-association, thereby driving phase separation and forming condensates. For example, Do et al. demonstrated that Y-shaped DNA nanostars undergo self-association via sticky ends to form phase-separated condensates [36]. Moreover, the partitioning and function of client DNA molecules within these condensates can be precisely controlled through toehold-mediated strand displacement. The formation of DNA-based condensates can also be modulated by environmental parameters such as temperature, salt concentration, and DNA concentration, enabling reversible transitions between liquid-like and gel-like states (Table 1).

## 3 Functionalization and recruitment strategies for client proteins

Client proteins are not strictly required for the assembly of MLOs, but they are essential for imparting specific biological functions to synthetic condensates. To date, various strategies have been developed to recruit diverse client molecules—including fluorescent proteins, regulatory factors, mRNAs, and enzymes—into synthetic MLOs, enabling customizable functionalization [1,4]. The most straightforward and widely used approach is direct fusion of IDPs to the N- or C-terminus of client proteins [8,10,12] (Fig. 1A). These IDPs mediate recruitment through multivalent interactions, driving the incorporation of clients into phase-separated condensates. However, this method may interfere with the native structure or function of client proteins due to the relatively large size of IDPs [4]. Although truncating IDPs can mitigate such effects, it often compromises the scaffold's capacity to form stable MLOs. For example, Song et al. demonstrated markedly higher recruitment efficiency for clients fused with full-length FUS domains compared to truncated FUS fragments [23].

To overcome the limitations associated with direct IDPs fusion, researchers have developed compact interaction motifs consisting of short peptide pairs that achieve efficient client recruitment while minimizing functional interference (Fig. 1B) [[37], [38], [39]]. A representative system is the RIAD/RIDD pair, derived from regulatory peptides of protein kinase complexes. RIAD (2.3 kDa) binds specifically to RIDD (6.7 kDa) to form a stable dimer both in vitro and in vivo [40,41]. By tagging client proteins with RIAD and scaffold IDPs with RIDD, client proteins can be recruited into synthetic condensates with remarkable efficiency. Compared to untagged proteins, RIDD-tagged proteins show an improved cargo protein enrichment (2-fold vs. 50-fold) [42]. Similarly, the SYNZIP1/SYNZIP2 small-peptide pair has shown superior recruitment efficiency over single IDP-based systems [39].

Multivalent modular domains offer another compact and tunable route for client recruitment. Classic folded domain–ligand pairs such as SH3/PRM and SUMO/SIM repeats drive selective client partitioning into condensates through their multivalency (Fig. 1C). Increasing scaffold valency (e.g., polySH3 or polySUMO arrays) or client valency (mono-, di-, or tri-valent PRM or SIM tags) generally lowers the *C*_sat_ while enhancing partition coefficients and selectivity [27,28]. Moreover, adjusting scaffold–client stoichiometry—for instance, providing an excess of polySUMO relative to polySIM—can switch the preferential enrichment of clients [14]. Orthogonal combinations (such as SUMO/SIM with SH3/PRM) further enable logic-like co-assembly and segregation at membranes or in the cytosol, achieving programmable co-localization or separation of endogenous receptors (e.g., EGFR, DR5, CXCR4) and thereby controlling signaling [29]. Compared with large IDP fusions, these folded-domain tags are more compact and orthogonal, and their valency and affinity can be finely tuned to yield robust, predictable recruitment and selectivity.

Beyond stable recruitment, dynamic control of client localization has been achieved by incorporating responsive modules (Fig. 1D). For instance, insertion of a TEV protease cleavage site between recruitment tags and client proteins allows protease-induced client release upon TEV expression [24]. Chemogenetic tools, such as FKBP-FRB domain fusions to scaffolds and clients, facilitate rapamycin-induced heterodimerization and reversible recruitment [6,13]. However, these methods typically support only a single cycle of recruitment or release. More recently, optogenetic and thermosensitive modules have been developed to enable reversible and repeatable control over client recruitment and release by modulating environmental cues such as light and temperature [6,8,21,43]. These advances provide powerful tools for engineering synthetic MLOs with enhanced functional versatility and dynamic control.

## 4 Assessing the physical properties of membraneless compartments

### 4.1 Morphological observations and spatial characterization

**Fig. 2:**
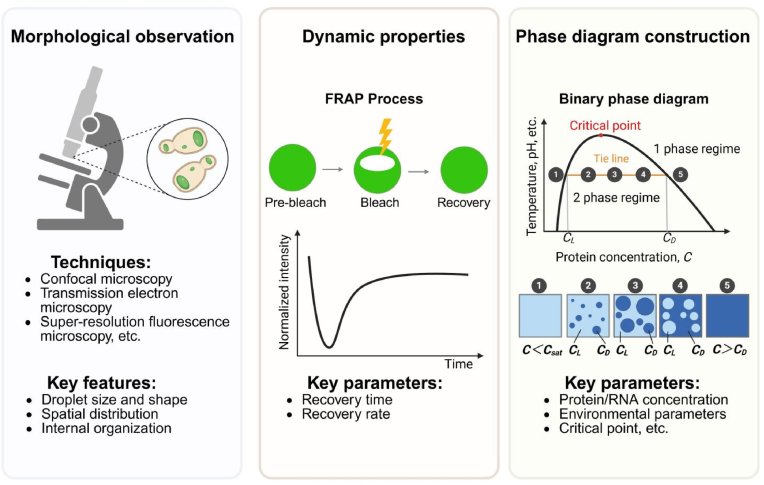
**Methods for characterizing membraneless compartments.** Left: Morphological observation. Middle: Dynamic properties quantified by FRAP. Right: Phase diagram construction. The coexistence curve (black) separates single-phase and two-phase regimes as functions of concentration (x) and conditions (y); the red dot marks the critical point. States on the same tie line (orange) demix into a dilute phase at *C*_*L*_ and a dense phase at *C*_*D*_; only their volume fractions change (lever rule). Colors indicate phase identity/state rather than absolute concentration values. Below *C*_*sat*_, the system remains in a single phase (panel 1). Panels 2–4 lie on the same tie line, sharing *C*_*L*_ and *C*_*D*_ but differing in their volume fractions. At concentrations exceeding *C*_*D*_, the system reverts to a single phase (panel 5). Note that *C*_*sat*_ the overall concentration at which droplets first appear under given conditions is not necessarily identical to *C*_*L*_.

A systematic assessment of the physical properties is essential for the rational design and deployment of synthetic condensates. Fluorescence microscopy is the primary tool to validate condensate formation and spatial organization in vitro and in vivo [3]. Confocal imaging of fluorescent protein-tagged scaffolds and clients reveals droplet presence, size distributions, and subcellular localization. In microbial cells, condensates typically appear as near-spherical foci and often localize to specific regions such as the cell center or poles [11,12] (Fig. 2). Such regular morphologies reflect surface tension-dominated shapes and suggest a dynamic equilibrium. By contrast, irregular droplet shapes, highly heterogeneous size distributions, or abundant unstable nanodroplets may indicate suboptimal conditions, including inappropriate expression levels, ionic strength, or pH [44]. Thus, morphology not only validates condensate formation but also guides optimization of parameters such as protein concentration, temperature, and ionic strength [45].

To gain more detailed insight into the internal organization of condensates, higher-resolution imaging techniques can be employed. Transmission electron microscopy (TEM) has been employed to visualize the ultrastructure, providing details on internal architecture that support identification of phase separation characteristics [35]. Super-resolution microscopy techniques enable molecular-level mapping of the spatial distribution and dynamic behavior of distinct components within droplets, providing deeper insights into their internal organization and compartmentalization [[46], [47], [48]]. Additional advanced imaging techniques, including Raman microscopy, Cryo-electron tomography (Cryo-ET), correlative light and electron microscopy (CLEM), and atomic force microscopy (AFM), have also been applied to observe and characterize membraneless compartments [26,[49], [50], [51]]. These complementary techniques allow the exploration of chemical composition, three-dimensional ultrastructure, and nanomechanical properties of condensates, thus enriching the multi-scale understanding of their physical characteristics. With the growing volume and complexity of microscopy data, deep learning algorithms have become valuable tools for automated droplet recognition and morphological analysis, greatly improving data processing efficiency and accuracy [52,53].

### 4.2 Dynamic behaviors and molecular mobility

The fluidity of synthetic condensates is a critical physical property that underpins their biological functionality and structural integrity. Fluorescence recovery after photobleaching (FRAP) is widely used to investigate the dynamic properties of condensates in vivo and in vitro [54]. In FRAP, a region of interest (ROI) is photobleached with a high-intensity laser, and the fluorescence recovery within the bleached region is monitored to analyze the diffusion kinetics of unbleached molecules entering the ROI (Fig. 2) [10,11,40]. In typical LLPS systems, short fluorescence recovery time and high recovery rate indicate rapid molecular exchange and liquid-like behavior. However, LLPS droplets may undergo dynamic maturation over time or under specific environmental conditions, which often results in a gradual loss of fluidity and transition toward gel- or solid-like states [16,55]. In microbial systems, it is recommended to use fresh cells in the logarithmic growth phase to ensure reliable experimental results and minimize the effects of droplet maturation [56].

Fluorescence loss in photobleaching (FLIP) complements FRAP by continuously bleaching a defined region while monitoring changes in fluorescence intensity in adjacent unbleached areas [57,58]. This method facilitates quantification of molecular exchange and the re-equilibration dynamics of fluorescent molecules across different condensate regions. For example, researchers combined FRAP and FLIP to characterize the dynamics of GFP-labeled PGL-1 protein in P granules. They observed that following photobleaching of half the granule, fluorescence recovered within seconds in the bleached portion while fluorescence diminished correspondingly in the unbleached region, indicative of rapid, diffusion-limited molecular exchange within the condensate [59].

For highly mobile molecules, fluorescence equilibration can occur within milliseconds. To capture the dynamic behavior of such rapidly diffusing molecules, the use of fast confocal microscopy systems (i.e., with the spinning disk configuration) is necessary [60]. In addition, several potential factors influencing the results must be considered. High-intensity laser illumination can induce phototoxicity in live cells, promote protein cross-linking, and disrupt protein activity, potentially resulting in artifactually static behavior. If such effects are observed, illumination conditions should be adjusted accordingly. Furthermore, prolonged laser exposure during time-lapse imaging may lead to global photobleaching of the sample. This issue can be addressed by incorporating control regions to correct for global photobleaching effects, thereby enhancing the accuracy and reliability of the experimental results [61].

### 4.3 Phase separation behaviors and phase diagram construction

Microscopy and FRAP reveal whether condensates form and how molecules move within them, while phase diagrams provide quantitative insights into the conditions under which phase separation occurs. This makes phase diagrams critical for understanding and controlling phase separation behaviors in vitro and in vivo [15,62]. Classical binary phase diagrams plot the concentration of a key component (e.g., protein or RNA) on one axis and a second parameter, such as temperature, salt concentration, or pH, on the other. The resulting curve, known as the binodal, encloses the two-phase region where dilute and dense phases coexist (Fig. 2) [4,15]. *C*_sat_ represents the threshold concentration at which phase separation begins, marking the boundary between the single-phase and two-phase regions. Within the two-phase region, the system separates into a dilute phase and a dense phase, which coexist in equilibrium. The equilibrium concentration of the dilute phase (*C*_L_) lies on the binodal and is typically slightly lower than the corresponding *C*_sat_ measured upon onset. At the apex of the binodal resides the critical point, beyond which (at higher temperature or pH, etc.) phase separation is not observed. Operating well within the binodal enables stable condensate formation critical for continuous bioengineering processes, whereas positions near the boundary favor dynamic assembly-disassembly suited for regulation. In multi-component systems, tie-lines connect bulk mixture compositions to phase-specific component ratios, guiding effective co-localization of enzymes, RNAs, or client proteins [63].

Phase diagrams in vitro are typically constructed by preparing samples with varying concentrations and buffer conditions in multi-well plates or microfluidic devices. After equilibration, phase separation onset is detected via light scattering or fluorescence microscopy [64,65]. The saturation concentration (*C*_sat_), or minimum concentration required to form droplets, is determined for each condition, producing the binodal curve when plotted. Advances in automated high-throughput imaging and analytical algorithms substantially improve precision and throughput while minimizing subjective bias [66]. Integrating quantitative fluorescence with mass spectrometry and theoretical modeling enables detailed characterization of multicomponent phase diagrams [[67], [68], [69]]. In vivo phase diagram construction requires precise control of intracellular conditions like protein concentration, pH, or multivalent interactions, using genetic, chemical, or optogenetic tools combined with advanced imaging techniques. For example, optogenetic systems like “Corelets” enable light-controlled oligomerization of IDPs, allowing the mapping of binodal boundaries in living cells through confocal microscopy and FRAP [19]. Similarly, a synthetic two-protein system in yeast, with tunable interaction affinity via point mutations, enabled high-resolution phase diagram construction by combining stochastic protein expression and fluorescence microscopy [70]. These in vivo approaches not only illuminate how cellular environments regulate phase separation but also support the design of synthetic condensates with controllable composition, dynamic behaviors, and specific functions.

In practical applications, the characterization of MLOs often requires the integration of multiple methods. Morphological observation confirms droplet formation, dynamic analysis reveals their fluidity, and phase diagram construction clarifies their environmental dependencies. By combining these approaches, a comprehensive understanding of their properties can be achieved, facilitating the optimization of their practical applications.

## 5 Application of phase-separated membraneless organelles in biotechnology

Over the past decade, researchers have successfully designed and constructed numerous phase-separated MLOs across diverse cellular and cell-free systems [4]. These engineered synthetic organelles, endowed with novel functions, have emerged as powerful tools for biotechnology [1,71,72]. Here, we present a categorized overview of their applications based on the latest advances in the field (Table 2).

**Table 2:** The construction and application of synthetic MLOs.

Scaffold	Client	Recruitment strategy	Response mode	System	Application	Reference
**Regulation of enzymatic activities and metabolic flux**						
A-IDPs	SfGFP, split GFP fragment, LacZ fragment	direct fusion,	temperature	*E. coli*	Sequestering proteins, modulating enzymatic activity	[22]
RGG	Idi, IspA	RIAD/RIDD interaction	N/A	*E. coli*	Enhancing the biosynthesis of α-farnesene	[40]
rCAG repeats (RNA)	GFP, Metabolic enzyme, β-gal fragments	RNA aptamer-protein binding (MS2/BoxB systems)	N/A	*E. coli*	Metabolic flux optimization, enzyme activity enhancement, translation buffering	[35]
DIX and DIX-like domains	EGFP, 2′-FL, LNT, and LNnT biosynthesis pathway enzymes	DIX–DIX heterotypic interaction, RIAD/RIDD interaction	N/A	*E. coli*	Boosting metabolic pathway flux, improving the production of desired chemicals	[73]
RGG	SfGFP, 2′-FL biosynthesis pathway enzymes	RIAD/RIDD interaction, SpyCatcher/SpyTag	N/A	*E. coli*	Enhancing the biosynthesis of 2′-fucosyllactose	[12]
SIDPs	GFP, 2′-FL, N-acetylmannosamine biosynthesis pathway enzymes	RIAD/RIDD interaction	N/A	*B. subtilis*	Enhancing the biosynthesis of desired chemicals and the translation specificity	[11]
FUSN-Cry2, FUSN-PixD/PixE	VioE, VioC (deoxyviolacein pathway)	light-induced clustering and dissociation	light	*S. cerevisiae*	Enhancing the production of target metabolites, Redirecting metabolic flux	[8]
RGG, WGR-1	GFP, mCherry, CFP, squalene and UA biosynthesis pathway enzymes	RIAD/RIDD interaction,SZ1/SZ2 pair	light	*S. cerevisiae*	Improving biosynthesis	[16]
**Control and probing of intracellular processes**						
RGG	Cdc24, Cdc5	TsCC(A)/TsCC (B) interaction	temperature	*S. cerevisiae*	Control of cell behavior	[13]
HOTag3/6	FKBP–FRB	FKBP/FRB interaction	rapamycin	HEK293	Visualizing dynamics of cell signaling	[74]
AG-PB1	N/A	Protein/protein (FKBP/FRB) interaction	rapamycin	HeLa, Cos-7, CHO–K1, HEK293	Visualizing protein interactions	[75]
PB1 and AG	mCherry, β-galactosidase, Vav2_cat_, SOS_cat_	FKBP/FRB interaction, LOV2/Zdk1 interaction	rapamycin, light	HeLa, NIH3T3, Cos-7	Controlling intracellular processes	[6]
**Recombinant protein expression and purification**						
BEAK-tag	Aβ-P3, TGFBIp1, TGFBIp2	direct fusion	N/A	*E. coli*	Efficient expression and purification of amyloidogenic peptides	[76]
RGG	EGFP, bpsA, melittin, and lactoferricin B	direct fusion	N/A	*C. glutamicum*	Reducing the toxicity of AMPs to host cells.	[10]
**Biomaterial**						
TDP-43 LC	Mfp5	direct fusion	N/A	In vitro	Bioadhesive materials	[77]
Recombinant MaSp2	N/A	N/A	N/A	In vitro	Protein fiber materials	[78]
Spidroin-SpyTag	CNTF, IGF1, OPN	SpyTag/SpyCatcher interaction	N/A	In vitro	Hydrogels	[79]
**Delivery Platforms**						
Phase-separating peptides (PSPs)	Protein, nucleic acid, peptide	Incubation	GSH	In vitro	Biomolecular delivery	[80]
Elastin-like polypeptides (ELPs)	FGF21	direct fusion	temperature	In vitro	Protein purification and delivery	[81]
ELP	GLP1	direct fusion	temperature	In vitro	Protein purification and delivery	[82]

### 5.1 Regulation of enzymatic activities and metabolic flux

Synthetic MLOs enable the spatial compartmentalization of enzymes and substrates within cells, offering precise spatiotemporal control over enzymatic reactions [8,13,83]. The unique physicochemical properties of LLPS generate condensates that serve as ideal microenvironments for synthetic biology and metabolic engineering [84]. Typically ranging from hundreds of nanometers to a few micrometers, these porous assemblies allow rapid influx and efflux of small molecules while accommodating high local concentrations of macromolecular catalysts. Moreover, environmentally responsive assembly and disassembly confer on-demand modulation of reaction rates [85].

By concentrating enzymes and substrates within specific regions, MLOs significantly increase their local concentrations, thereby promoting enzymatic activities. For example, the recruitment of SUMOylating enzymes and substrates into synthetic condensates enhances the rate of SUMOylation by up to 36-fold [86]. Similarly, co-compartmentalization of kinases and their substrates within MLOs can greatly enhance phosphorylation reactions [87]. This enhancement can be attributed to the proximity of enzymes and substrates, which reduces the free energy required for reactions and increases the frequency of enzyme-substrate interactions. MLOs can also improve reaction efficiency by decreasing the Michaelis constant (Km) of substrates or increasing the catalytic constant (Kcat) of enzymes [22,88]. Nevertheless, for substrates with low Km values, their enrichment within the condensates may lead to substrate inhibition, ultimately reducing reaction rates [86]. In addition, the physicochemical properties of condensates can significantly impact enzymatic reactions, high viscosity or gel-like structures may hinder diffusion rates and decrease enzymatic activity [16,31].

Beyond single-step reactions, MLOs offer a modular strategy to optimize multi-enzyme pathways [8,12,73]. By clustering multiple enzymes within a single condensate, MLOs reduce loss of labile intermediates, suppress side reactions, and limit the accumulation of toxic byproducts. For example, synthetic disordered proteins (SIDPs) have been used to construct biomolecular condensates that recruit key enzymes for 2′-fucosyllactose (2′-FL) biosynthesis in *B. subtilis*, achieving titers up to 681.6 mg/L [11]. In microbial cell factories, metabolic pathways often include complex branching points where multiple reactions compete for the same intermediates. The dynamic assembly and disassembly of pathway-specific enzymes within condensates enables a redistribution of intracellular metabolic flux. For instance, in the biosynthetic pathway of deoxyviolacein (DV), the enzymes VioE and VioC compete for the intermediate PTDV to produce pre-violacein and violacein, respectively. Synthetic MLOs can redirect metabolic flux and mitigate pathway competition [8,41]. Utilizing the PixELL optogenetic system, the assembly and disassembly of enzymes can be flexibly controlled. Under dark conditions, VioE and VioC coalesce within condensates to maximize DV production, whereas blue light triggers PixELL-mediated disassembly and redirects PTDV toward pre-violacein and violacein [8].

### 5.2 Control and probing of intracellular processes

**Fig. 3:**
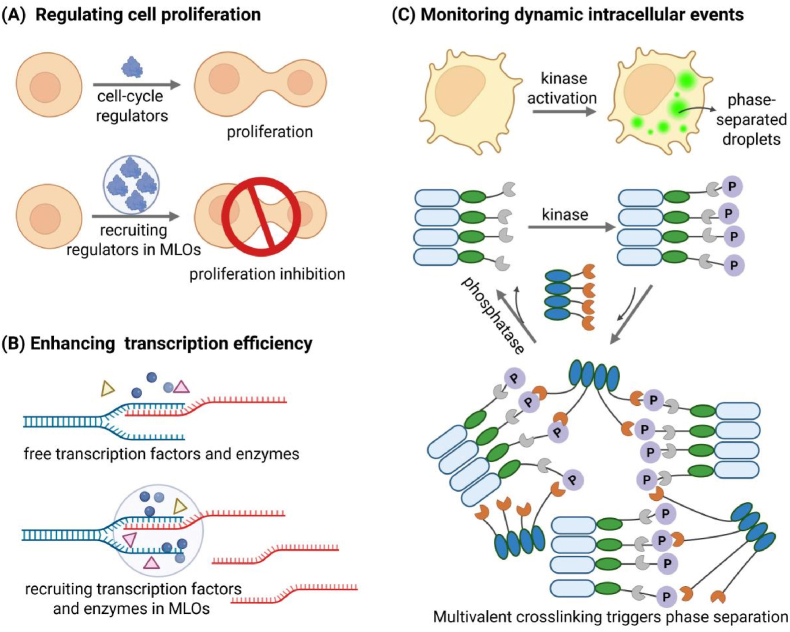
**Applications of synthetic MLOs in controlling and probing intracellular processes.** (A) Regulating cell proliferation; (B) Enhancing transcription efficiency; (C) Real-time visualization of kinase activity using a phase separation–based reporter (SPARK).

Precise regulation of intracellular processes is essential for understanding cellular functions and engineering biological systems. Conventional methods like genetic mutations or small molecule inhibitors often suffer from off-target effects, limited reversibility, and low spatial resolution [89]. Recently, synthetic biomolecular condensates have emerged as powerful tools to control cellular activities by spatially compartmentalizing specific molecules and biochemical reactions (Fig. 3A). For example, Garabedian et al. developed a condensate platform using a trivalent RGG-domain scaffold that forms stable droplets. By incorporating high-affinity dimerization tags, this system efficiently recruits endogenous target proteins, including Cdc24 and Cdc5, into condensates. Over 90 % of these proteins are sequestered, thereby preventing their substrate interactions and effectively regulating critical cellular processes, including polarity establishment, cell proliferation, and cytoskeletal organization [13]. Incorporation of thermo- or light-responsive domains further facilitates dynamic release and functional recovery of target proteins, enabling rapid, reversible modulation of cellular phenotypes [19,21,71].

Beyond modulating cellular behavior, LLPS has also been leveraged to enhance gene transcription efficiency and translation specificity. By fusing IDPs derived from the FET family, such as FUS and TAF15, to transcriptional activation domains within the CRISPR-SunTag system, researchers have engineered phase-separated condensates at specific genomic loci. These condensates effectively recruit key transcriptional machinery components, including BRD4 and RNA polymerase II, resulting in significantly improved transcription elongation and endogenous gene activation in mammalian cells and in vivo models [9] (Fig. 3B). Similarly, Yu et al. constructed artificial organelles that concentrate orthogonal tRNAs, tRNA synthetases, and target mRNAs within condensates. This compartmentalization significantly improved the efficiency of incorporating non-canonical amino acids (ncAAs), while minimizing cross-reactivity with the host translation system. Applying this system to optimize biosynthesis of N-acetylmannosamine (ManNAc) resulted in a 75 % increase in product output [11].

Moreover, LLPS-based MLOs provide valuable platforms for probing and monitoring dynamic intracellular events, owing to their ability to amplify molecular signals and respond rapidly to cellular stimuli [90]. The SPARK (separation of phases-based activity reporter of kinase) probe, for instance, detects kinase activity in real time via phase separation triggered upon kinase activation. Composed of a fluorescent protein, an oligomeric coiled-coil domain, and kinase-specific substrate peptides, SPARK enables sensitive, reversible, and straightforward tracking of kinase signaling in cells and live organisms. Compared to fluorescence resonance energy transfer (FRET) sensors, SPARK demonstrates larger fluorescence changes and enhanced suitability for in vivo imaging [74] (Fig. 3C). LLPS has also been applied to visualize protein-protein interactions (PPIs). The Fluoppi system, developed by Watanabe et al., utilizes PPI-dependent droplet formation to detect interactions with high clarity [75]. By fusing potential interacting partners to complementary domains, fluorescent droplets form exclusively upon protein interaction, generating a sharp “on-off” signal with a high signal-to-noise ratio. Using Fluoppi technology, researchers have successfully analyzed various PPIs and assessed drug-induced PPI blocking effects.

### 5.3 Recombinant protein expression and purification

**Fig. 4:**
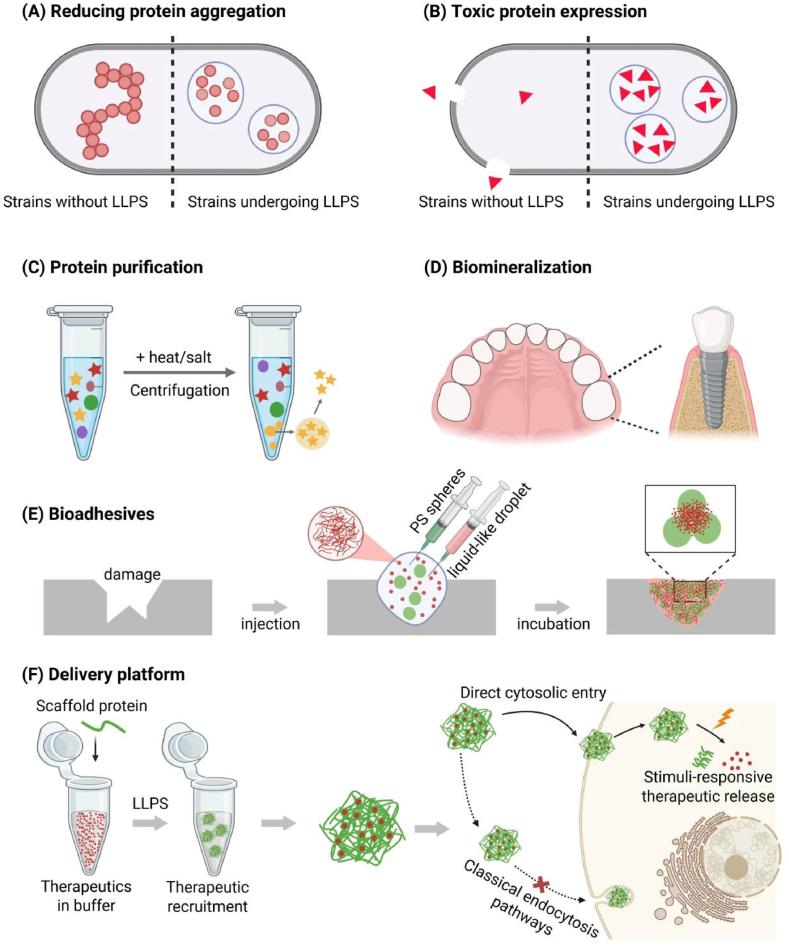
**Applications of synthetic MLOs.** (A) Synthetic MLOs to reduce early protein aggregation; (B) Sequestration of proteins to mitigate toxicity in host cells; (C) LLPS based recombinant protein purification; (D) LLPS-based biomineralization for premolar implants; (E) Schematic of Teflon substrate repair: condensate and polystyrene spheres are co-injected into the damaged region; after 12 h at 4 °C, the aged condensate adheres the spheres to the surface; (F) Schematic of delivery via MLOs formed by phase-separating peptides: they enter the cytoplasm directly, bypassing classical endocytosis.

Recombinant protein expression and purification are essential processes in life science research and biotechnology, where the activity and purity of proteins are pivotal for successful functional assays, structural characterization, and industrial use. Nonetheless, expressing certain proteins, especially those with high hydrophobicity, strong aggregation tendencies, or intrinsic toxicity, remains challenging [91,92]. Such proteins often exhibit low solubility, form insoluble inclusion bodies, or impair host cell viability. Recently, LLPS-driven MLOs have emerged as a powerful strategy to address these challenges. MLOs formed via LLPS create a protective microenvironment that can substantially reduce early protein aggregation and prevent unfavorable chemical modifications (Fig. 4A). For instance, Gabryelczyk et al. developed an LLPS-based fusion tag, termed BEAK-tag, which enabled efficient expression of amyloidogenic peptides in *E. coli* [76]. The BEAK-tag induces intracellular droplet-like MLOs that shield target peptides from premature aggregation, significantly enhancing their solubility and structural homogeneity. Similarly, our recent work demonstrated that LLPS-induced MLOs can spatially segregate proteins to mitigate toxicity toward host cells. By triggering LLPS in *C. glutamicum*, we achieve successful expression of antimicrobial peptides (AMPs) while reducing their cytotoxic effects [10] (Fig. 4B).

LLPS also offers a simple and cost-effective alternative for recombinant protein purification [93]. Affinity chromatography, while widely used in laboratory-scale workflows, is often limited in large-scale production due to the high cost of resins and operational expenses. LLPS-based purification exploits reversible phase transitions to concentrate and separate proteins without relying on chromatographic steps. For example, BEAK-tag condensates form dynamically under low pH during purification, facilitating target protein enrichment. Subsequent trypsin cleavage releases the protein, yielding high-purity products without additional chromatography [76]. To further simplify purification and avoid the need for enzymatic cleavage, alternative LLPS scaffolds have been developed. Elastin-like polypeptides (ELPs), for example, undergo phase separation triggered by temperature or salt concentration changes, enabling efficient protein enrichment [94] (Fig. 4C). By incorporating self-cleaving intein systems into ELPs scaffolds, tag removal can be achieved under mild conditions through intein-mediated autoprocessing, eliminating the need for external enzymes [95]. Additionally, affinity ligand-ELPs fusion systems that incorporate specific affinity ligands (e.g., Protein A) provide an efficient solution for purifying tag-free proteins, such as monoclonal antibodies. In these systems, target proteins are captured by the ligand and then released by modulating pH, salt, or temperature to disrupt the interaction [96]. These systems eliminate the need for enzymatic cleavage or chromatographic steps, offering a scalable and cost-efficient solution for producing high-purity recombinant proteins.

### 5.4 Functional biomaterials engineering

LLPS has emerged as a versatile molecular self-assembly strategy for engineering functional biomaterials with hierarchical architectures, dynamic functionalities, and biomimetic properties [97]. LLPS-mediated protein condensates can stabilize amorphous biomineral precursors, which, when integrated with structural matrices like collagen or nanocellulose, guide ordered crystallization [98]. This biomineralization strategy allows the fabrication of hierarchical composites that mimic the mechanical gradients found in natural tissues like bone and teeth, thereby enhancing mechanical strength and durability [99] (Fig. 4D). LLPS also inspires the design of bioadhesive materials capable of strong adhesion under challenging conditions, including underwater or wet environments. For example, Mussel foot proteins (Mfps), rich in 3,4-dihydroxyphenylalanine (DOPA) residues, form liquid-phase condensates within acidic vesicles that undergo pH-triggered metal ion crosslinking to yield strong adhesive plaques [100]. Inspired by this natural mechanism, recombinant Mfp-based adhesives were developed and demonstrated strong adherence to diverse substrates such as polytetrafluoroethylene and polyethylene terephthalate [77] (Fig. 4E). Similarly, sandcastle worm proteins undergoing LLPS-driven condensate formation have been engineered with polyelectrolytes like hyaluronic acid to create bioadhesive hydrogels effective in wound closure and tissue repair, showing excellent biocompatibility and adhesion in wet environments [101].

Furthermore, LLPS plays a critical role in fabricating high-performance protein fibers. Spider and silkworm silk proteins are stored as concentrated liquid condensates within specialized glands; environmental triggers such as pH gradients, kosmotropic salts, or mechanical shear induce liquid-to-solid transitions (LSTs) that generate semicrystalline fibers with exceptional mechanical properties [102]. Inspired by this process, recombinant spider silk and regenerated silkworm silk proteins have been engineered to undergo LLPS under physiological conditions, forming stretchable liquid condensates that solidify into fibers upon controlled pH shifts or shear forces [78]. Microfluidic technologies further enhance control over the spinning process by simulating shear forces and pH gradients, enabling the production of fibers with hierarchical structures and superior mechanical performance [103]. Additionally, LLPS has shown great potential in developing hydrogels that rapidly gel via liquid-to-solid phase transitions and effectively bind various bioactive molecules. For instance, LLPS-based hydrogels formed from recombinant spider silk proteins combined with SpyTag/SpyCatcher chemistry provide sustained release of neurotrophic factors such as ciliary neurotrophic factor (CNTF) [79]. When injected into ocular tissues, these hydrogels prolong signaling pathway activation and promote axonal regeneration. Their excellent mechanical properties, injectability, and biocompatibility make them promising materials for applications in nerve repair, protein drug delivery, and tissue engineering [79].

### 5.5 Cytosolic delivery for biomolecules

Synthetic MLOs have emerged as promising delivery platforms due to their unique physicochemical properties and dynamic microenvironments generated via phase separation [[104], [105], [106]] (Fig. 4F). These condensates can protect therapeutic agents from degradation and enable stimuli-responsive release profiles. Compared to conventional chemically synthesized polymer carriers, protein-based LLPS systems offer superior biocompatibility and safety, as they are composed of natural amino acids [4]. Moreover, these protein materials can be precisely engineered through genetic techniques to tailor their physicochemical properties, allowing precise control over drug release rates and targeting capabilities [105]. In addition, LLPS-based systems can directly penetrate cell membranes without relying on traditional endocytosis pathways, enabling efficient cytosolic delivery while avoiding lysosomal degradation [105]. LLPS-based systems can undergo phase transitions triggered by various external stimuli, such as temperature, glutathione (GSH), pH, or light, thereby forming drug reservoirs that improve stability and enable sustained release. For example, fusing glucagon-like peptide-1 (GLP-1) with ELPs prolongs its half-life and enhances therapeutic efficacy via temperature-induced phase separation [82]. Furthermore, LLPS platforms can be engineered to achieve targeted drug accumulation at pathological sites. In tumor therapy, this approach markedly increases local drug concentrations while minimizing systemic toxicity, offering novel strategies for drug delivery in complex pathological environments [107].

LLPS-based delivery systems can also overcome limitations of traditional carriers that often rely on single-drug loading by enabling simultaneous delivery of multiple biomacromolecules. For instance, a recent study developed a redox-responsive phase-separating peptide (HBpep-SP) that forms synthetic MLOs capable of efficiently delivering all three major CRISPR/Cas9 gene-editing formats: plasmid DNA (pDNA), messenger RNA (mRNA), and Cas9 ribonucleoprotein complexes (RNPs) [80]. This platform demonstrated superior delivery efficiency and gene-editing efficacy compared to commercial reagents. Importantly, it enables precise intracellular cargo release through a GSH-triggered smart response, significantly enhancing therapeutic safety and effectiveness [80].

Despite these advantages, the practical application of LLPS-based delivery systems still faces challenges. For instance, delivery efficiency can be influenced by the complexity of in vivo environments, and the currently limited diversity of LLPS-triggering mechanisms may restrict the precision of targeted release [108]. Additionally, high design costs of LLPS-based systems and the technical challenges of large-scale production need to be addressed [106]. Nevertheless, with continuous advancements in biomaterials science and engineering, LLPS-based delivery platforms are expected to overcome these bottlenecks, providing an innovative, flexible, and efficient solution for drug delivery.

## 6 Conclusion

In recent years, synthetic MLOs have been successfully constructed in various microorganisms and eukaryotic cells using both natural and engineered intrinsically IDPs. These synthetic MLOs exhibit dynamic behaviors, selective molecular enrichment, and concentration effects, establishing them as innovative tools for enzyme activity regulation, biological process monitoring, protein expression and purification, and functional biomaterials (Table 2). The integration of regulatory modules, such as light-responsive, temperature-sensitive, and pH-responsive elements, has further enhanced their controllability and expanded their application potential in synthetic biology and biotechnology [1,4].

Despite these advancements, practical applications of synthetic MLOs still face several challenges. For instance, the physicochemical properties of MLOs often differ between in vitro and in vivo contexts, complicating their predictability and stability. [10,12,40]. Additionally, the risk of liquid-to-solid phase transitions over time can compromise their functionality, and there is a need for more orthogonal, tunable scaffolds to ensure compatibility across diverse biological systems [31]. While certain scaffolds, such as RGG or PB1-AG fusion constructs, have demonstrated low cytotoxicity in short-term studies, their long-term effects on cellular health require systematic investigation [6,40]. Moreover, precisely organizing multiple enzymes at optimal ratios within a single MLO and understanding the diffusion behavior of cargo molecules remain significant technical challenges [4,109]. Looking ahead, emerging technologies offer promising solutions to overcome these hurdles. Advances in AI-assisted protein design, high-resolution imaging, and innovative synthetic biology tools are expected to facilitate the development of more stable and responsive MLOs systems [37,66,[110], [111], [112]]. For example, AI-driven approaches could enable the rational design of next-generation scaffolds with enhanced phase separation properties and reduced cytotoxicity [53,111]. Additionally, high-throughput screening methods could accelerate scaffold optimization and improve large-scale production efficiency [103,113].

LLPS-based synthetic MLOs are anticipated to play an increasingly important role in both fundamental research and industrial applications. They hold significant promise for revolutionizing metabolic engineering, drug delivery, and precision medicine by providing dynamic microenvironments that can be precisely controlled and customized for specific needs. These developments not only offer exciting opportunities to deepen our understanding of biological phenomena but also pave the way for transformative innovations in biotechnology and medicine.

## CRediT authorship contribution statement

**Manman Sun:** Writing – review & editing, Writing – original draft, Funding acquisition, Conceptualization. **Alex Xiong Gao:** Writing – review & editing, Software, Formal analysis, Conceptualization. **Bin Ye:** Writing – review & editing, Conceptualization, Formal analysis. **Yimeng Zhao:** Software, Methodology, Formal analysis. **Rodrigo Ledesma-Amaro:** Writing – review & editing, Supervision, Conceptualization. **Jin Gao:** Software, Formal analysis. **Peng Wang:** Writing – review & editing, Supervision, Funding acquisition.

## Declaration of competing interest

The authors declare that they have no known competing financial interests or personal relationships that could have appeared to influence the work reported in this paper.

## Data Availability

All data supporting the conclusions of this study are derived from the cited literature, and no new datasets were generated.
